# Antisepsis and the Country Physician

**Published:** 1889-03

**Authors:** G. Walter Barr

**Affiliations:** Bridgeport, Ill.


### ANTISEPSIS AND THE COUNTRY PHYSICIAN.

BY G. WALTER BARR, M. D, BRIDGEPORT, ILL.

The warning by Dr. J. M. Taylor, in the REPORTER of December 8, 1888, regarding antiseptics incites me to contribute briefly my experience on the same subject. I practise in the couutry, over the worst roads in the nation, and when I leave home have no idea whether I will have a case of placenta prævia, fractured femur, typhoid fever, or hypermetropic headache before I return. Under such circumstances the practitioner must be physician, surgeon and inventor combined. Our city cousins have no idea of our trials and emergencies, but I verily believe that the situation has advantages, as a discipline, over the conveniences of town practice. With experience as a student in several medical colleges, the best training I ever received was the teaching of Professors Gross and Brinton at the Jefferson College, where we saw little intricate mechanism or perfected instruments, but heard much about improvised helps " trunkmaker's board."

Having used the best possible judgment in discriminating between new remedies and fads, the country practitioner is liable to be shy of furors like Bergeon's treatment, but to take up and hold fast that which is good. The greatest God-send we have received for many a day is antisepsis. No one has a more correct idea of the word than the country doctor. He cannot carry the apparatus of a hospital and must somehow create the condition extemporaneotsly with all kinds of means. Consequently he can not follow a formula, but must know exactly the end to be obtained and the principles to be followed in attaining it. The most convenient antiseptic for our class is bichloride of mercury, because of its very small bulk in ratio to it efficacy. Ammonium chloride is always carried for cough-mixtures, and this dissolves the bichloride in rainwater. Enough gauze for one dressing has a small bulk, and so has a suitable quantity of iodoform. With these in his case, the country doctor takes whatever is at hand and operates antiseptically. When he insists on having a disc instead of a tin cup for his solution, his learning is wondered at; when he amputates some fingers and tells the patient not to touch the dressing until he returns in a week or so the old nurse thinks he is daft; and when he takes off the dressings and shows a healed scar he is regarded as either an angel or a wizard. But aside from the carnal benefits to his purse, antisepsis saves him a world of trouble. I dress an operation-wound antiseptically and tell the nurse to notify me in case the secretions wet through the bandage, or any fever or unplesant odor is noticed. Then I return when I am next in the neighborhood to remove the dressings. This is somewhat better than riding half the night in the rain. Of course some cases cannot be so treated; but still antisepsis is a great boon. I will not discuss the question of the value of antisepsis *per se*. The country doctors have waited until the matter is past the experimental stage; if they will learn the fundamental principles of the art and use it after their usual customs, they will never discard it.—*Med. and Surgical Reporter*.

